# Attitudes and misconceptions towards sharks and shark meat consumption along the Peruvian coast

**DOI:** 10.1371/journal.pone.0202971

**Published:** 2018-08-29

**Authors:** Rocío López de la Lama, Santiago De la Puente, Juan Carlos Riveros

**Affiliations:** 1 Institute of Resources, Environment and Sustainability–IRES, The University of British Columbia, Vancouver, Canada; 2 Laboratorio de Estudios en Biodiversidad–LEB, Universidad Peruana Cayetano Heredia, Lima, Peru; 3 Institute for the Oceans and Fisheries, The University of British Columbia, Vancouver, Canada; 4 OCEANA Peru, Lima, Peru; Stockholms Universitet, SWEDEN

## Abstract

Misconceptions, lack of knowledge, and negative attitudes towards sharks act as barriers preventing actions required to tackle threats to shark populations, limiting the success of global shark conservation initiatives. Peru, a major player for the international trade of shark products, recently approved the ‘National Action Plan for the Conservation and Management of Sharks, Rays and Chimaeras’ (PAN-Tib); a guiding document for conservation initiatives aimed at these fishes. Within PAN-Tib, the assessment of Peruvians’ current knowledge and attitudes towards sharks is listed as a research priority. Between June and October 2016, 2004 Peruvians were surveyed along the coast to characterize their (i) shark meat consumption patterns, and (ii) knowledge and attitudes towards sharks. Results suggest that shark meat consumption is extended, but not necessarily frequent, and higher in the northern regions of the country. However, 77.5% of shark meat consumers were unaware that they had eaten sharks. Although 57.6% of the participants recognized that sharks are present in Peruvian waters, only 19.4% of the surveyed population was capable of naming at least one local shark species. Moreover, Peruvians have very negative attitudes towards sharks. They fear them and view them as man-eaters, despite this, no shark attacks have ever been reported in the country. These results highlight the need to: (i) encourage sustainable shark meat consumption, and (ii) promote communication campaigns aimed at increasing knowledge about sharks, and their importance as a source of employment and food for coastal communities, as for the national economy.

## Introduction

Despite some recent improvements on the development of sustainable shark fisheries around the world [1], the success of shark conservation initiatives has been limited by factors including but not limited to their negative public image [2–6]. Mass media has reinforced inaccurate knowledge about this group of species for decades, depicting them as vicious man-eating murderers that need to be culled for our safety [5–9]. This in turn has led to their social marginalization, the legitimization of permissive harvesting rules, and the lack of action in favour of their recovery [2,8].

For example, shark coverage by US and Australian newspapers has focused almost exclusively on shark attacks and their negative effects on humans, neglecting other pressing issues such as their current conservation status or ecological roles [9]. In northeastern Brazil, low levels of understanding of the situation faced by sharks and its implications on human well-being, coupled with the negative attitudes towards sharks among coastal citizens, have resulted in non-engagement and disregard for conservation actions seeking to safeguard shark populations [4]. Similarly, negative public perceptions and lack of knowledge also played important roles in limiting community engagement for shark conservation initiatives in the UK [3].

Attitudes, knowledge and behaviours are closely related, influencing actions and decision-making [10–12]. Studies suggest that educational interventions and other knowledge building activities can increase positive attitudes [7]. Strengthening these factors may contribute to pro-environmental behaviours [4,7,13]. Additionally, increased public awareness and understanding of environmental problems can help build the capacities required to solve them [2,8,14].

Peru’s marine ecosystems are among the most productive in the world [15]. They harbour a high fish diversity (~1070 spp., [16]), including 66 shark species [17]. Peru is currently a major player in the international trade of shark products [18]. The shark fisheries provide significant sources of employment and revenue for Peruvians [19], despite declining trends in landings over time [20].

At least 32 shark species are caught by small-scale fishers using gillnets or longlines in Peru [20, 21]. Their combined landings for the 2006–2015 period averaged 8,000 tonnes year^-1^ [22], and only six species accounted for 98% of them. These were: blue sharks (*Prionace glauca*), shortfin makos (*Isurus oxyrinchus*), smooth hammerheads (*Sphyrna zygaena*), humpback smooth-hounds (*Mustelus whitneyi*), Pacific angelsharks (*Squatina californica*) and thresher sharks (*Alopias sp*.) [20, 23].

Nonetheless, official records of shark landings and trade are commonly misleading as there is evidence of species misidentification [23] and shark species are generally grouped under generic categories for reporting purposes [20]. Most shark landings are labelled as ‘tollo’, a generic name originally used only for hound sharks (Triakidae spp., [24]). Fishers and retailers use ‘tollo’ as a common name for all sharks (or ‘tiburones’ in Spanish). For example, the common name for blue shark in Peru is ‘tiburón azul’, but it is common to find blue shark meat in markets labelled as ‘tollo azul’, ‘tollo’ or just ‘azul’ [25], eliminating any direct reference to sharks. The practice of renaming seafood is not restricted to Peru [26]. For instance, the Spiny dogfish (*Squalus acanthias*), a demersal shark species, is commonly sold as ‘rock salmon’ or ‘hass’ in the USA, and used for popular dishes like fish and chips, deceiving most consumers [26, 27].

Sharks are facing their largest crisis over their 420-million-year history mainly because of fishing for their fins and meat [1]. These species are highly vulnerable to overfishing given their life history traits which include: slow growth rates, late age of maturity, long gestations periods, low fecundity, and long life spans [3, 9].

In a context where losses to marine biodiversity are occurring on a global scale, public support for shark conservation is critical to foster research, improve management and promote sustainable use [8]. In order for shark conservation campaigns to be successful, inaccurate information and negative stereotypes about these species must be replaced by a factual narrative highlighting sharks’ regulatory role in marine ecosystems and their contribution to local and global economies [1,2,4].

In 2014, the Peruvian government developed the National Action Plan for the Conservation and Management of Sharks, Rays and Chimaeras (PAN-Tib) [28]. This document highlights the need to generate information for communication and education campaigns aimed at raising awareness and increasing community engagement with shark management and conservation efforts. However, very little is known about Peruvians’ current knowledge and attitudes towards this group of fishes. This information is needed to tailor campaigns for key demographics and assess their effectiveness. Hence, the main objectives of this paper were to explore and characterize: (i) coastal Peruvians’ shark meat consumption patterns, as well as their (ii) knowledge and attitudes towards sharks.

## Methods

### Study area

**Fig 1:**
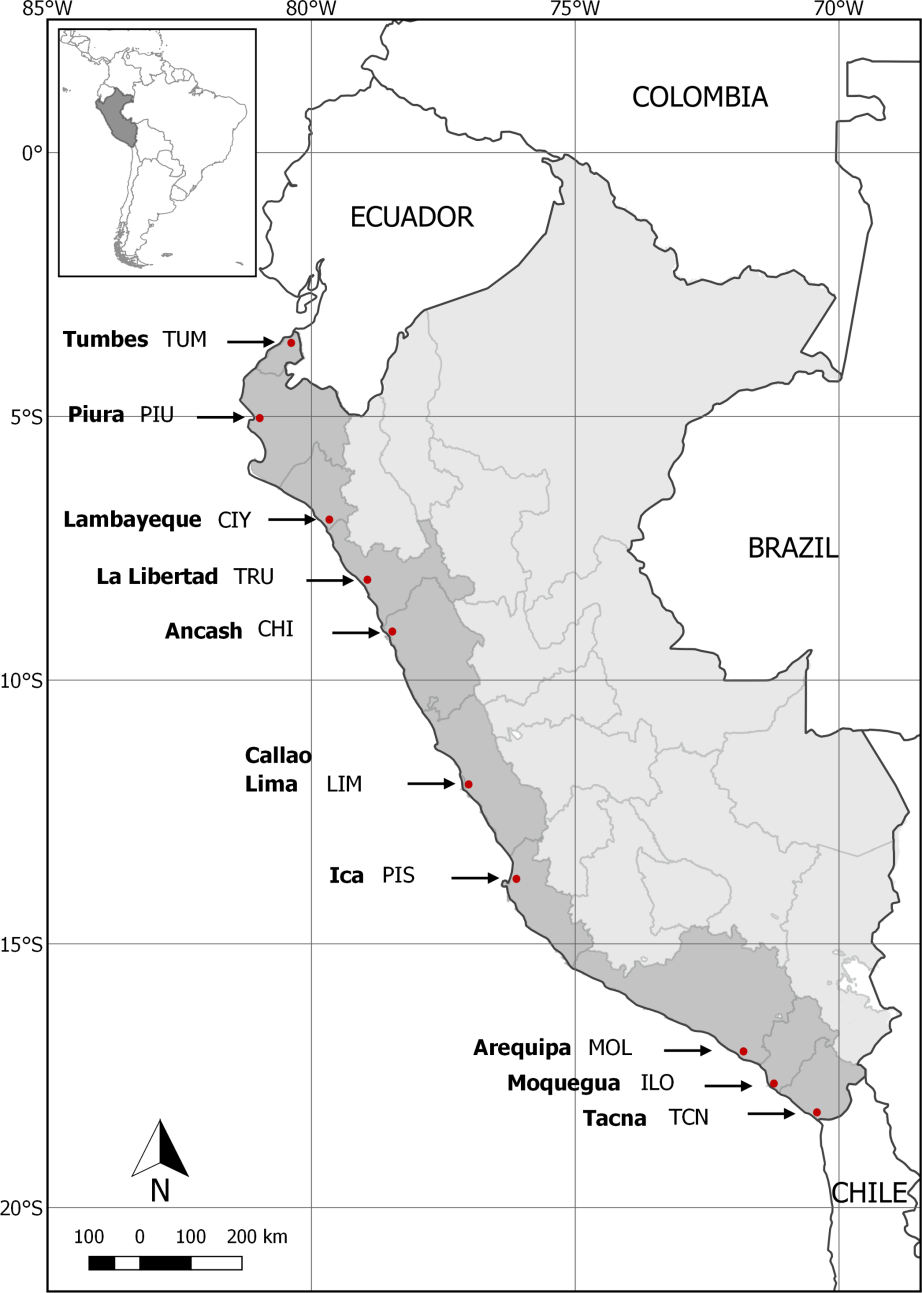
Peruvian coastal regions, highlighting the cities were the surveys were implemented. Coastal regions include: Tumbes (surveyed city Tumbes, TUM), Piura (surveyed city Piura, PIU), Lambayeque (surveyed city Chiclayo, CIY), La Libertad (surveyed city Trujillo, TRU), Ancash (surveyed city Chimbote, CHI), Lima (surveyed city Lima, LIM), Callao (not surveyed), Ica (surveyed city Pisco, PIS), Arequipa (surveyed city Mollendo, MOL), Moquegua (surveyed city Ilo, ILO) and Tacna (surveyed city Tacna, TCN).

One city from each of Peru’s 11 coastal regions was selected for data collection purposes. All cities included in the study (a) had more than 10 thousand inhabitants, (b) were located less than 60 km from the coastline, and (c) are politically and economically important for their respective regions (Fig 1). As the Callao Region is located 15 km away from Lima’s city centre, and surrounded by the Lima Region, surveys conducted in Lima are assumed to be representative of Callao as well.

### Survey characteristics

Voluntary participants (i.e., adult residents of the selected cities) completed an anonymous short survey with 10 questions. Surveys lasted approximately 20 minutes and were conducted by professional pollsters between June and October of 2016 (S1 Table). Pollsters covered the cities’ most important public spaces (e.g., shopping malls, pedestrian thoroughfares, and public plazas), selecting participants at random.

This research was approved by the Ethics Committee of the Cayetano Heredia University, Lima, Peru (Project No. 101775). Participants gave their verbal consent and were informed that they could stop the survey at any point.

#### Survey validation and minimum sampling size

Before the survey was conducted in the selected cities, it was validated by interviewing 85 people in Lima during March 2016. As 93% of the participants of this preliminary survey believed that sharks were present in Peruvian waters, we used this proportion to estimate a statistically significant sample size per city (Eq 1) that would guarantee an approximate 95% confidence level in the responses [29].

$$n=\frac{pq{\left(1.96\right)}^{2}}{d^{2}}$$ Eq 1

Where: *n* is the sample size, *p* is the proportion of the population that correctly held the pre-assessed belief (i.e., shark presence in Peru), *q* = 1−*p*, and *d* is the degree of precision (d = 0.05 denotes a margin of error of 5%). Following this equation, a minimum sample size of 100 participants per city was estimated.

#### Data processing and analysis

Descriptive statistics, correlations and statistical tests were computed using R (Ver. 3.4.0). All variables were tested for normality (Shapiro-Wilk 1965) and did not follow a normal distribution. Thus, non-parametric tests were used to identify statistically significant differences between subsets of the surveyed population. Average values are presented followed by the corresponding standard deviation ($\bar{x}\pm \sigma$).

### Levels of consumption

Participants were classified into five categories according to their claims regarding shark meat consumption:

- Regular consumers: people who eat shark meat at least twice a month;
- Occasional consumers: people who eat shark meat once a month;
- Infrequent consumers: people who almost never eat shark meat or do not recall the frequency at which they do;
- Former consumers: people who used to eat shark meat but not anymore; and
- Non-consumers: People who do not eat and have not eaten shark meat.

The difference between Regular and Occasional consumers was determined based on the results of the Peruvian National Household Survey [30]. Coastal consumers of seafood eat on average 0.81 kg of seafood per month [30], which is equivalent to three servings per month [25].

### Attitudes scores and profiles

Attitudes about sharks were explored via *word association*, a qualitative method commonly applied in psychology [31]. The premise of this technique is that the first words that come to mind -in relation to an object or concept- are the most relevant for the person [32, 33]. Participants mentioned up to three words, which were used to build a shark-related vocabulary. The words were classified and scored as negative (-1 point), neutral (0 points) and positive (1 point). Then they were grouped into eight sub-categories, highlighting the type of information they carried:

Positive words:

- Benefits to humans, which included words that denote knowledge of the direct or indirect benefits that sharks, their fisheries and trade generate for Peruvians (e.g., food, work, tourism);
- Positive feelings, which included words that reference emotions aligned with pro-environmental sentiments (e.g., respect, awe, admiration); and,
- Positive traits, which included adjectives or anthropomorphic attributes of sharks that are regarded as desirable by society (e.g., grand, pretty, smart).

Neutral words:

- Ecological and biological knowledge, which included words that indicate basic knowledge of shark ecology and biology (e.g., aquatic, predator, carnivore); and,
- Miscellaneous, which included words not directly associated with sharks and that could not be classified as part of the other categories (e.g., movie, dolphin, beach).

Negative words:

- Negative outcomes of human-shark interactions, which included words that reference lethal and traumatic outcomes of shark attacks (e.g., blood, death, screams);
- Negative feelings, which included words that reference emotions that typically result from the belief that sharks are a threat to humans (e.g., fear, desperation, tears); and,
- Negative traits, which included adjectives or anthropomorphic attributes of sharks that are rejected by society (e.g., dangerous, murderer, evil).

An Individual Attitude Score (IAS) was computed for each participant by averaging the values of the words they mentioned. A General Attitude Score (GAS) was calculated for all of coastal Peru, by averaging all IAS. Values for all attitude scores range between -1 and +1.

## Results

A total of 2004 surveys were conducted along the Peruvian coast, exceeding the minimum sample size in all cities. Participants were on average 40 ± 14 years of age (Range: 18–93 years); 56% self-identified as women; and 67% had undertaken higher education either at universities or technical institutions (S2 Table).

The following sections describe Peruvian shark meat consumption patterns, as well as the knowledge and attitudes towards sharks at the national level. Plots summarizing results per surveyed city are included in S1–S6 Figs.

### Shark meat consumption

Shark meat is popular amongst coastal Peruvians, as 72.4% of the surveyed population (n = 1451) claimed to eat or have eaten sharks. However, shark meat can be purchased using different names in the local seafood markets. Most shark meat consumers (76.2%, n = 1106) claim to only have eaten ‘tollo’, whilst a very limited proportion of them claims to have exclusively eaten ‘tiburón’ (1.7%, n = 24). The remaining consumers stated that they had eaten shark meat under both names (22.1%, n = 321).

Assuming that people who claim to have eaten ‘tollo’ but not ‘tiburón’ ignore that ‘tollo’ is in fact a commercial name used for multiple shark species, then only 22.5% of the ‘tollo’ consumers were aware of their shark meat consumption.

Given that 28.8% of the surveyed population had never eaten ‘tollo’ and that 12.5% no longer consumes it, then 57.8% of the survey participants were current ‘tollo’ consumers. Most current consumers were regular consumers (37.6%), followed by infrequent (32.7%) and occasional consumers (29.6%).

**Fig 2:**
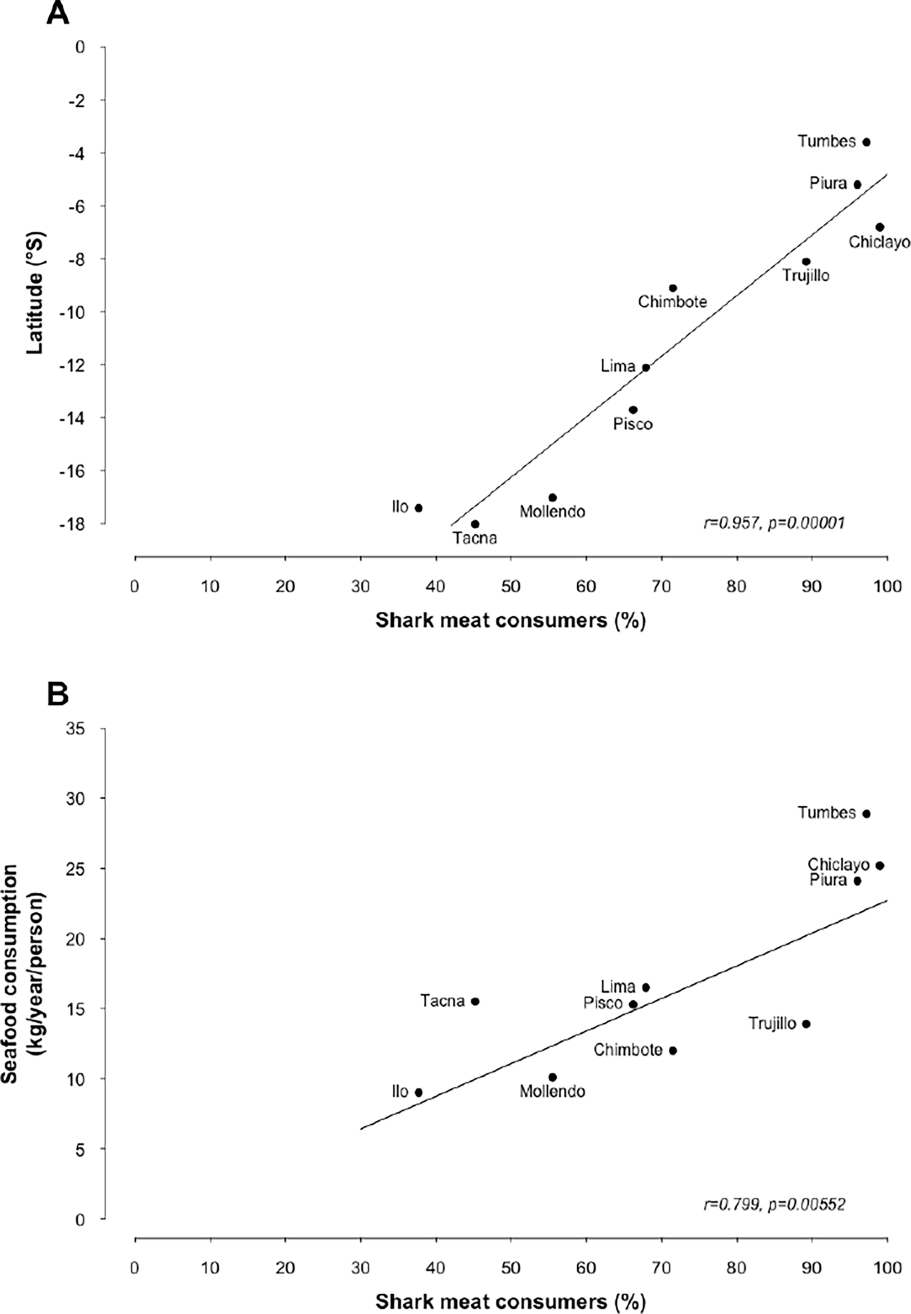
Proportion of shark consumers per city. Proportion of consumers as a function of (A) latitude, and (B) the *per capita* consumption of seafood in 2015.

It is important to note that shark meat consumers represented a higher proportion of the surveyed population in the northern regions of Peru and significantly decreased towards the southern regions (r = 0.957, p = 0.00001*) (Fig 2A). Similarly, shark meat consumers represented a larger proportion of the population in cities where the *per capita* seafood consumption was higher (r = 0.799, p = 0.00552*) (Fig 2B).

**Fig 3:**
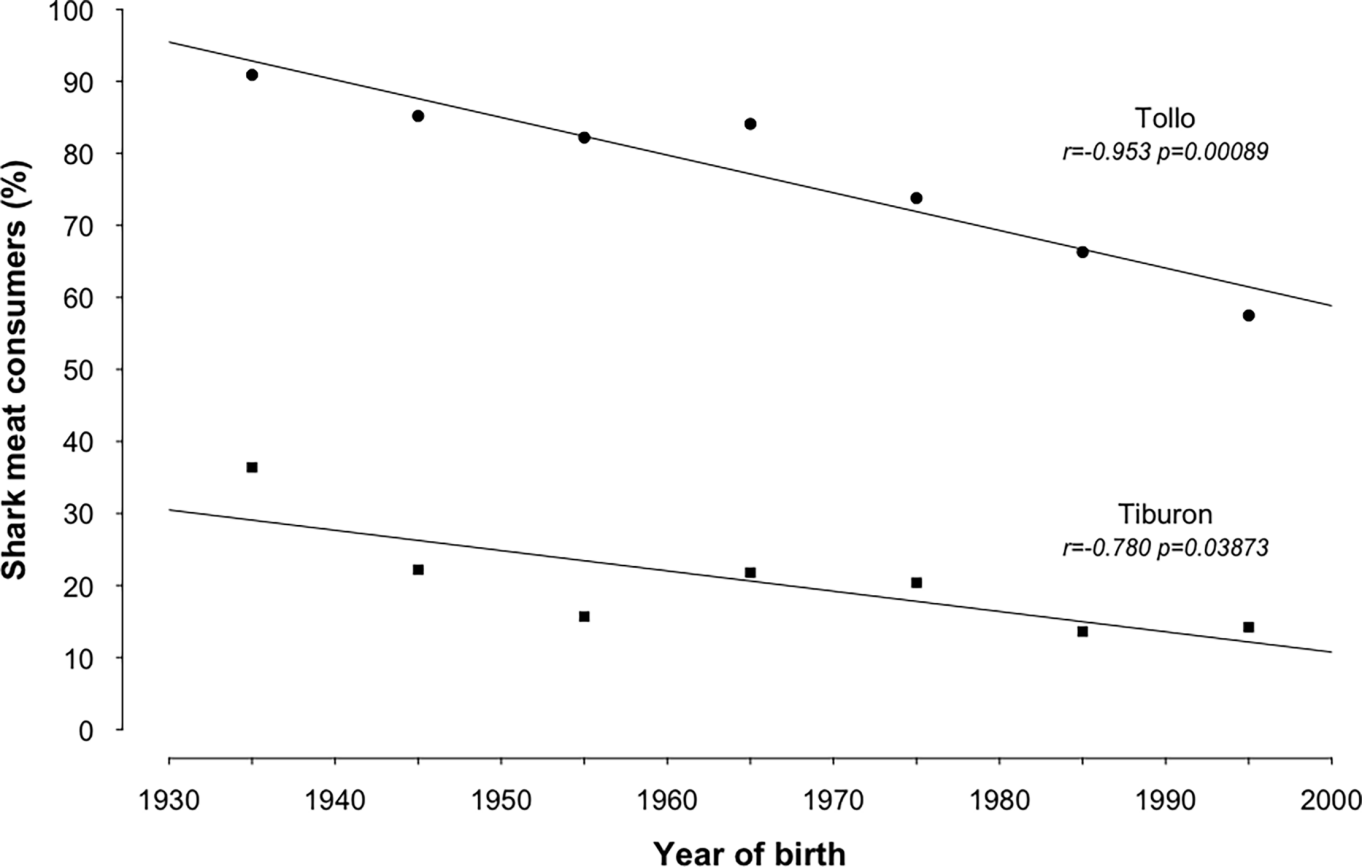
Proportion of shark meat consumers as a function of the decade of their birth. Shark meat consumers are segregated as people who claim to eat or have eaten ‘tollo’ and ‘tiburón’.

Statistically significant negative correlations were found between the decade in which participants were born and (a) the proportion that alleged to eat ‘tollo’ (r = -0.953, p = 0.00089*), as well as (b) the proportion that claimed to eat ‘tiburón’ (r = -0.780, p = 0.03873*) (Fig 3). No relevant differences were identified between the proportion of the population that claims to consume ‘tollo’ and/or ‘tiburón’ and the level of education or gender.

### Knowledge about sharks

More than half of the surveyed population (57.6%) knew that sharks inhabit Peruvian waters. A significant negative correlation was found between the decade in which participants were born and the proportion who knew that sharks were found in Peru (r = -0.903, p = 0.005*). For instance, a participant born in the 1990s was 25.2% less likely to know that sharks were found in Peru in comparison to someone born in the 1940s.

Additionally, less than half of the participants that recognized that sharks were present in Peru (46.7%, n = 1155) could name at least one shark species by common name. Of this subset of the surveyed population 72% provided one name, 22.8% provided two names and 5.2% provided three names. However, when expressed as proportions of the whole surveyed population, only 19.4% named one shark species that lived in Peruvian waters, 6.1% named two, 1.4% named three and 73.1% named none. Participants recognized a total of nine common names of sharks present in Peru: blue shark (27.3%), smooth-hound (18.1%), hammerhead shark (16.8%), great white shark (14.9%), shortfin mako shark (14.5%), tiger shark (4.2%), sharptooth smooth-hound (1.9%), whale shark (1.8%), and thresher shark (0.6%).

### Attitudes towards sharks

**Fig 4:**
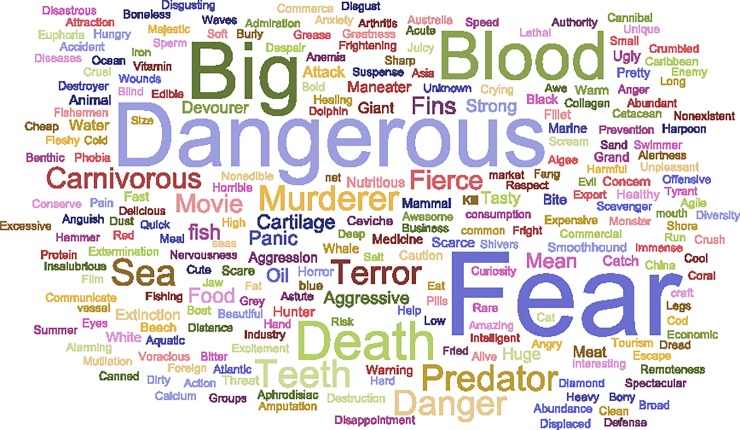
Word cloud highlighting words mostly frequently associated with sharks by coastal Peruvians. Words were translated from Spanish. Font size is proportional to word frequency.

The surveyed population mentioned 5772 words associated with sharks (i.e., total word count, TWC), forming a shark-related vocabulary of 354 different words (S3 Table). Only fourteen words were repeated by more than 100 participants, constituting 66.3% of all given words (Fig 4). These were: fear (12.6%), dangerous (9.5%), big (8.3%), blood (6.9%), death (5.1%), teeth (3.4%), sea (3.2%), predator (3.0%), murderer (2.9%), danger (2.8%), terror (2.8%), carnivorous (2.1%), fierce (1.9%) and movie (1.8%). At the sub-category level, the most mentioned words corresponded to ‘Ecological and Biological Knowledge’ (26.3%), followed by ‘Negative feelings’ (22.3%), and ‘Negative traits’ (19.1%) (Table 1).

**Table 1:** Words most frequently associated with sharks by coastal Peruvians.

Category	Sub-Category	Most common words
Positive words (12.6%)	Benefits to humans (9.4%)	Fins, food, cartilage, oil, tasty
	Positive traits (2.3%)	Strong, fast, grand, pretty, astute
	Positive feelings (0.9%)	Awe, awesome, respect, curiosity
Negative words (55.4%)	Negative outcomes of human-shark interactions (13.9%)	Blood, death, attack, aggression, bite
	Negative traits (19.1%)	Dangerous, murderer, fierce, mean, aggressive
	Negative feelings (22.3%)	Fear, danger, terror, panic, horror
Neutral words (32%)	Ecological and biological knowledge (26.3%)	Big, teeth, sea, predator, carnivorous
	Miscellaneous (5.7%)	Movie, white, blue, beach, whale

Percentages correspond to the relative contribution of each category and sub-category to the total word frequency.

Overall, most words mentioned by participants had negative connotations (55.4% of the TWC), whilst neutral (34.3% of the TWC) and positive (10.3% of the TWC) words were less frequent. This distribution skewed the attitude scores towards negative values, resulting in a General Attitude Score (GAS) for Peru of: –0.43 ± 0.41.

### Individual attitudes scores—IAS

IAS were not significantly correlated with participant’s age (r = -0.122, p = 0.326). Female participants had significantly lower IAS than those of males (p<0.0001*), and participants with university degrees had higher IAS than those with technical studies (p = 0.046*) and secondary studies (p = 0.005*). Although consumers and non-consumers of ‘tollo’ had similar IAS, consumers of ‘tiburón’ had significantly higher IAS than non-consumers (p = 0.0002*).

Additionally, regular and occasional consumers of ‘tollo’ had lower IAS than former (p = 0.008* and p = 0.001*, respectively) and infrequent consumers (p = 0.012* and p = 0.001*, respectively). Finally, participants who knew that ‘tollo’ is a common name for shark or who knew that sharks are found in Peruvian waters had significantly higher IAS than their less informed counterparts (p = 0.001* and p<0.0001*, respectively).

## Discussion

### Shark meat consumption

Consumption of shark meat has been part of the coastal Peruvian culinary tradition for at least 10,000 years [34–36]. From the results of this survey, shark consumption is still common along the coast, but not necessarily frequent. Seven in ten coastal Peruvians claimed to eat or have eaten shark meat, but only two in ten claimed to be regular consumers. Yet, shark consumption could be more common and/or frequent due to widespread seafood mislabelling [25, 26], as sharks might be sold under other fish names to improve their marketability [37].

Shark meat consumption was not homogeneous along the Peruvian coast. Seafood consumption is heavily influenced by fish availability in local markets, price accessibility for consumers, and culinary tradition [38]. Thus, it is not surprising that cities found in the northern regions of Peru contained a larger proportion of shark meat consumers (Fig 2), and particularly ‘regular’ shark meat consumers. Northern Peru is characterized by higher *per capita* consumption of seafood [39], greater diversity of commercial shark species [21], and a tradition of shark meat consumption [40].

Our results show that the majority of consumers were unaware that ‘tollo’ meat is shark meat, with only 22.5% of all ‘tollo’ consumers knew that they were eating sharks. Most shark species caught in Peruvian waters are sold under this generic name, albeit the actual landings of ‘tollo’ (Triakidae spp.) have declined over time [20, 25, 41]. This is evidence that the market is masking and diluting signals from marine ecosystems [42].

It is worth noting that the proportion of ‘aware’ shark meat consumers was not higher in segments of the population that had greater academic training, nor differed with age. This suggests that the disconnection between shark meat consumers and sharks transcends education and generational barriers. Nonetheless, more people were aware that ‘tollo’ was a shark name in regions where sharks represented a larger proportion of the total local landings of fish for human consumption. For instance, Ilo had the largest proportion of ‘aware’ shark meat consumers, as it is the leading landing site for oceanic pelagic sharks in Peru [20], and sharks represent 26.5% of the regional landings for direct human consumption (S4 Table). This would suggest that a heightened regional visibility of shark fisheries and their landings could improve consumer awareness.

### Knowledge about sharks

This survey revealed that coastal Peruvians’ general knowledge about sharks is low, varied little between cities and did not improve with academic development.

Peru is home to 9% of all living chondrichthyan species reported around the world [17], and sharks represent 6.2% of Peru’s marine fish diversity [24]. Nonetheless, six in ten coastal Peruvians were aware of shark’s presence in Peruvian waters, and only three of them could name one or more shark species. Participants mentioned a total of nine common names for sharks, representing only 13.6% of the total shark species found in Peru [17].

Additionally, Peruvian small-scale fishers target sharks for their meat and fins [20]. Sharks, as they flowed from the sea to final consumers across the seafood value chain, generated US$50 million and 4600 jobs in Peru in 2009 [19]. Nonetheless, words related to the direct or indirect benefits that sharks generate for Peruvians were limited (i.e., <10% of the TWC), and references to activities, such as tourism, which are beneficial and non-lethal to sharks were negligible. Similarly, coastal Peruvians’ biological and ecological knowledge of sharks is limited to them being carnivorous active predators, with large body sizes and teeth. No references to sharks’ supporting and regulatory roles in marine ecosystems were recorded in this study. However, sharks are important biological controllers of cephalopods in Peruvian waters (e.g., smooth hammerheads on the jumbo squid [43]).

### Misconceptions and negative attitudes towards sharks

In Peru, sharks continue to suffer from a negative public image, as more than half of the words mentioned by the surveyed population had negative connotations. The extensive use of words like ‘fear’, ‘dangerous’, ‘terror’, ‘blood’, ‘death’ and ‘murderer’, suggest that Peruvians see sharks as menacing deadly man-eaters. However, no shark attacks (fatal or otherwise) have ever been reported in Peruvian waters and only 15 shark attacks have been reported by Chile and Ecuador combined between 1900 and 2016 [44].

Nevertheless, local newspapers routinely cover stories of shark attacks and share dramatic videos of such encounters recorded elsewhere. As seen in other contexts, mass media has a strong influence on how citizens perceive sharks, mainly by perpetuating the notion that sharks are dangerous and a threat to human survival [2, 5–9]. Given the negative connotation of the majority of words mentioned by coastal Peruvians, it is not surprising that the GAS was negative. However, it is worth noting that the IAS of people with university level education, as well as those who knew that sharks were present in Peruvian waters, and/or that ‘tollo’ is used as a generic name for sharks, were less negative. This would suggest that there is a positive link between knowledge and attitudes, as seen in other countries and contexts [4, 7,14].

Finally, positive words beyond benefits to humans were rare, accounting for 3.2% of the TWC. Positive concepts and feelings inspired by sharks are almost non-existent, representing a challenge for securing citizen’s engagement in future shark conservation campaigns [3]. Peruvians still feel more fear and danger when thinking about sharks than awe and wonder, unlike other countries (e.g., UK and Australia) where a transition from ‘seeking protection from’ to ‘seeking protection for’ sharks has already started [3, 6].

### Reconnecting coastal citizens and sharks

Misconceptions, lack of knowledge, and negative attitudes towards sharks act as barriers preventing actions required to tackle threats to shark populations worldwide [2–9]. These factors could also be limiting shark management and conservation initiatives in Peru. As citizens are unaware of the presence, consumption, vulnerability and conservation status of sharks in Peruvian waters, they are unlikely to demand improvements in fisheries management and enforcement [2–4].

Knowledge and attitudes towards sharks were low and negative, irrespective of education level, age, or gender, across all surveyed cities; highlighting a high level of disconnection between coastal citizens and sharks. This could be explained by two factors: poor environmental education and limited opportunities for taking part in positive experiences with sharks. For example, a recent review of the academic curricula of public schools across the country found that learning objectives’ coverage of global and local marine topics was very limited, with no specific mentions of sharks [45]. Moreover, there are no aquariums in Peru featuring local shark species, and shark dives are not accessible to most of the population. Thus, the only people in frequent and direct contact with living sharks in Peru are fishers, and their interactions are not necessarily positive.

Personal experiences are key factors that influence environmental attitudes and motivate personal engagement and pro-environmental behaviours [46]. In the Peruvian context, positive direct experiences with sharks could be fostered by highlighting their role as seafood. Peruvians love seafood and traditional ingredients possess high cultural values [37, 47]. If Peruvian consumers were aware that they eat and enjoy eating sharks, and that these species are threatened, they would be in a better position to demand sustainable shark fisheries. This would require: (i) reducing unaware shark meat consumption, perhaps through a ‘one name, one fish’ policy [48]; (ii) raising consumer awareness about shark species diversity, their ecological roles and conservation status; and, (iii) raising awareness of sustainable shark meat consumption practices such as: avoiding threatened species, respecting minimum landing sizes and seasonal closures.

Yet, conservation campaigns should not be limited to shark meat consumers. These efforts should be framed within nationwide education campaigns targeting the general public, fishers, companies, and government institutions, as proposed by the National Plan of Action for the Conservation of Sharks, Rays and Chimeras of Peru [28].

### Methodological considerations

This paper presents a novel method for exploring attitudes towards sharks within the discipline of marine conservation. However, it is important to consider two issues that might affect result interpretation.

First, the categories used to group the results of the word association test, and the way they were scored, were based on the direct connotation of the listed words. Let’s take for example the words ‘teeth’, ‘fear’ and ‘movie’ to explore our rationale. ‘Fear’ directly reveals a negative feeling resulting from the thought of sharks. Hence, it was grouped under a negative sub-category. However, ‘teeth’ might suggest fear towards sharks or simply a distinct physical attribute of sharks; whilst ‘movie’ might be associated with action films with negative depictions of sharks (e.g., Jaws) or a documentary seen during Shark Week. Thus, words such as ‘teeth’ or ‘movie’ were grouped under neutral categories. As a result of these aggregations, it is possible that attitudes towards sharks might be more negative than currently presented.

Second, although our data gathering efforts exceeded the minimum sample size required for statistical representation of the coastal cities, we found that the surveyed population had a higher level of education than the national average [49]. This could be explained by the fact that the surveyed coastal cities are the regional capitals which tend to congregate universities and other post-secondary learning centres, as well as the jobs that demand the highest levels of academic qualifications [49].

Based on these issues, we suggest for future studies to: (i) ask participants to provide some context for the words they mentioned during the word association test; (ii) extend the survey to other important coastal cities and rural areas; and, (iii) apply a stratified random sample at each surveyed location.

## Conclusions

Consumption of shark meat is common along the Peruvian coast, although most shark consumers ignore the fact that they are eating sharks due to the use of the generic name ‘tollo’. Awareness of shark meat consumption does not vary according to education or age, suggesting that the disconnection between sharks and coastal citizens transcends education and generational barriers. Furthermore, people’s knowledge about shark was limited to physical characteristics (e.g. big, teeth, fin) and ignored the high diversity of shark species in Peru. In the same line, perceptions towards sharks remain highly negative, projecting strong negative feelings from the participants. Therefore, misconceptions, lack of knowledge and negative attitudes towards sharks in Peru could be acting as barriers for conservation and management initiatives; highlighting the need of Peruvians to reconnect with sharks.

## Supporting information

S1 Table Structure of questions of the survey. (PDF)

S2 Table General description of the surveyed population. The acronyms used in this table stand for: No.—Number of participants; M—male; F—female; Elem.—Elementary; H-S—High-school; Tech.—Technical; and Uni.—University. In some cities, sex ratios and/or education levels do not sum to 100% as some participants failed to provide this information.
(PDF)

S3 Table Shark-related vocabulary. Composed by the words mentioned by the surveyed population when asked: *What words come to mind when you hear the word ‘sharks’*?
(PDF)

S4 Table Total fish landings and shark landings caught by the small-scale fishing fleets of Peru. (PDF)

S1 Fig Proportion of shark meat consumers per city. (A) Consumers who eat sharks under the ‘Tiburon’ (grey bars) and ‘Tollo’ (blue bars) common names, and (B) ‘Tollo’ consumers who are aware that ‘Tollo’ is a generic name for sharks (i.e., ‘Conscious’ shark meat consumers).
(PDF)

S2 Fig Distribution of ‘tollo’ consumers per city as a function of their frequency of shark meat consumption. Shark meat consumers were categorized into: Regular consumers (REG), Occasional consumers (OCC), Unusual consumers (UNU) and Former consumers (FOR)
(PDF)

S3 Fig Proportion of the surveyed population per city that knows that sharks are present, maybe present, or are not present in Peruvian waters. The proportion of participants that did not answer this question are included in the N/A category.
(PDF)

S4 Fig Capacity to mention shark names by participants who ascertained that sharks are present in Peruvian waters, segregated city. N references the total number of mentions, μ references the average number of words mentioned per city and σ references its standard deviation.
(PDF)

S5 Fig Radar plots showing the relative importance of the eight different categories that constitute each city’s attitude profile. Each radar consists of four concentric octagons that extend from the origin. Each level denotes a 10% increase in the frequency of the words per category, where the origin marks a score of 0% and the outermost octagon of 40%. Acronyms in the figure stand for: BH: Benefits to humans; M: Miscellaneous; NT: Negative traits; NO: Negative outcomes of human-shark interactions; NF: Negative feelings; EBK: Ecological and biological knowledge; PT: Positive traits; PF: Positive feelings.
(PDF)

S6 Fig Distribution of the individual attitude scores per city. μ references the average attitude score per city and σ references its standard deviation.
(PDF)

S1 Database Survey answers of the 2004 participants about shark perceptions and consumption patterns. The questions of the survey (Sheet 1), relevant variables and answers (Sheet 2), and words related to sharks provided by participants (Sheet 3 to 5) are available in the database, each participant has a unique Identification Code.
(XLSX)
