# Supplementary material for: Attitudes and misconceptions towards sharks and shark meat consumption along the Peruvian coast
Source: PLoS One. 2018 Aug 29;13(8):e0202971. doi: 10.1371/journal.pone.0202971 (PMC6114843; doi:10.1371/journal.pone.0202971)
Supplement: S1 Table — (PDF) [file pone.0202971.s001.pdf]

S1 Table. Structure of questions of the survey.

No.	Questions	Answer options
1	Year of birth	Open ended question
2	Gender	Male<br>Female<br>Non-binary
3	Place of birth	Open ended question
4	Highest completed level of education	Elementary<br>High-school<br>Technical<br>University<br>NA
5	Have you ever eaten ‘tollo’?	Yes<br>No<br>Do not know
6	With what frequency do you eat ‘tollo’?<br><br>*Only asked if the participant answered ‘Yes’ in the previous question*	Once a week<br>Twice a month<br>Once a month<br>Hardly ever<br>Do not recall<br>Used to, but not anymore
7	Do you believe that sharks are found in Peruvian waters?	Yes<br>No<br>Do not know
8	What species of sharks are found in Peruvian waters?<br><br>*Only asked if the participant answered ‘Yes’ in the previous question*	Open ended question
9	Have you ever eaten sharks?	Yes<br>No<br>Do not know
10	What words come to mind when you hear the word ‘shark’?	Open ended question
