# Supplementary material for: Attitudes and misconceptions towards sharks and shark meat consumption along the Peruvian coast
Source: PLoS One. 2018 Aug 29;13(8):e0202971. doi: 10.1371/journal.pone.0202971 (PMC6114843; doi:10.1371/journal.pone.0202971)
Supplement: S2 Table — The acronyms used in this table stand for: No.—Number of participants; M—male; F—female; Elem.—Elementary; H-S—High-school; Tech.—Technical; and Uni.—University. In some cities, sex ratios and/or education levels do not sum to 100% as some participants failed to provide this information. (PDF) [file pone.0202971.s002.pdf]

S2 Table. General description of the surveyed population. The acronyms used in this table stand for: No. - Number of participants; M - male; F - female; Elem. - Elementary; H-S - High-school; Tech. - Technical; and Uni. - University. In some cities, sex ratios and/or education levels do not sum to 100% as some participants failed to provide this information.

City	No.	Age (in years)			Sex ratio		Education level			
		Average	S.D.	Range	M	F	Elem.	H-S	Tech.	Uni.
Tumbes	178	45.7	14.4	19-89	53.5%	43.5%	12.5%	45.5%	22.5%	18.5%
Piura	201	40.5	12.6	20-74	45.5%	53.5%	3.5%	44.0%	17.5%	35.0%
Chiclayo	199	40.2	13.7	19-72	34.2%	64.3%	4.0%	20.1%	36.7%	37.7%
Trujillo	204	38.0	10.9	19-78	36.2%	63.3%	2.5%	8.0%	37.2%	51.3%
Chimbote	200	38.0	15.3	19-83	56.4%	41.5%	2.1%	20.1%	26.9%	50.9%
Lima	234	41.6	14.8	19-93	45.3%	50.7%	7.0%	39.3%	36.3%	14.4%
Pisco	201	40.4	12.4	19-76	37.8%	62.7%	1.5%	21.9%	24.9%	50.7%
Mollendo	200	35.0	13.6	19-79	42.6%	57.4%	3.7%	22.3%	33.0%	41.1%
Ilo	199	41.4	12.8	19-84	27.0%	72.5%	3.9%	25.0%	25.0%	44.1%
Tacna	188	39.3	15.0	18-79	43.8%	53.9%	9.6%	25.8%	25.8%	40.4%
