# Supplementary material for: Attitudes and misconceptions towards sharks and shark meat consumption along the Peruvian coast
Source: PLoS One. 2018 Aug 29;13(8):e0202971. doi: 10.1371/journal.pone.0202971 (PMC6114843; doi:10.1371/journal.pone.0202971)
Supplement: S3 Table — Composed by the words mentioned by the surveyed population when asked: What words come to mind when you hear the word ‘sharks’? (PDF) [file pone.0202971.s003.pdf]

S3 Table. Shark-related vocabulary (SRV). Composed by the words mentioned by the surveyed population when asked: *What words come to mind when you hear the word 'sharks'?*

Words in Spanish	Words in English	Category	Sub-category	Frequency
Miedo / Temor	Fear	Negative	Negative feelings	728
Peligroso	Dangerous	Negative	Negative traits	547
Grande	Big	Neutral	Ecology and biological knowledge	480
Sangre	Blood	Negative	Negative outcomes	396
Muerte	Death	Negative	Negative outcomes	297
Diente / Dientes	Teeth	Neutral	Ecology and biological knowledge	194
Mar	Sea	Neutral	Ecology and biological knowledge	183
Depredador	Predator	Neutral	Ecology and biological knowledge	174
Asesino	Murderer	Negative	Negative traits	168
Peligro	Danger	Negative	Negative feelings	164
Terror	Terror	Negative	Negative feelings	159
Carnívoro	Carnivorous	Neutral	Ecology and biological knowledge	124
Salvaje / Feroz	Fierce	Negative	Negative traits	109
Película	Movie	Neutral	Miscellany	104
Aletas	Fins	Positive	Commercial benefits	95
Pez, Peces , o Pescado	Fish	Neutral	Ecology and biological knowledge	67
Malo	Mean	Negative	Negative traits	67
Alimento/ Comida	Food	Positive	Commercial benefits	65
Cartílago	Cartilage	Positive	Commercial benefits	61
Pánico	Panic	Negative	Negative feelings	59
Aceite	Oil	Positive	Commercial benefits	54
Fuerte	Strong	Positive	Positive traits	49
Agresivo / Agresividad	Aggressive	Negative	Negative traits	48
Devorador	Devourer	Negative	Negative traits	41
Rico / Sabroso	Tasty	Positive	Commercial benefits	35
Enorme	Huge	Neutral	Ecology and biological knowledge	34
Carne	Meat	Positive	Commercial benefits	33
Ataque	Attack	Negative	Negative outcomes	28
Extinción	Extinction	Neutral	Ecology and biological knowledge	27
Gigante	Giant	Neutral	Ecology and biological knowledge	26
Come gente	Man-eater	Negative	Negative traits	26
Cazador	Hunter	Neutral	Ecology and biological knowledge	25
Nutritivo	Nutritious	Positive	Commercial benefits	23
Agresion	Aggression	Negative	Negative outcomes	22
Feo	Ugly	Negative	Negative traits	22
Pesca	Catch	Positive	Commercial benefits	21

S3 Table. Shark-related vocabulary (SRV). *(Continued)*

Words in Spanish	Words in English	Category	Sub-category	Frequency
Blanco	White	Neutral	Miscellany	21
Animal	Animal	Neutral	Ecology and biological knowledge	20
Mordedura / Mordida / Muerde	Bite	Negative	Negative outcomes	20
Escaso	Scarce	Neutral	Ecology and biological knowledge	19
Horror	Horror	Negative	Negative feelings	17
Susto	Scare	Negative	Negative feelings	17
Agua cálida	Warm water	Neutral	Ecology and biological knowledge	16
Amenaza	Threat	Negative	Negative feelings	16
Veloz	Fast	Positive	Positive traits	16
Medicina	Medicine	Positive	Commercial benefits	14
Azul	Blue	Neutral	Miscellany	14
Playa	Beach	Neutral	Miscellany	14
Advertencia / Alerta	Warning	Negative	Negative feelings	14
Precaución / Caultela	Caution	Negative	Negative feelings	14
Filete	Fillet	Positive	Commercial benefits	13
Cuidado / Preocupación	Concern	Negative	Negative feelings	13
Ceviche	Ceviche	Positive	Commercial benefits	11
Agua	Water	Neutral	Ecology and biological knowledge	11
Ballena	Whale	Neutral	Miscellany	11
Imponente	Grand	Positive	Positive traits	11
Saludable / Salud	Healthy	Positive	Commercial benefits	10
Especie marina	Marine	Neutral	Ecology and biological knowledge	10
Negro	Black	Neutral	Ecology and biological knowledge	10
Tollo	Smooth-hound	Neutral	Ecology and biological knowledge	10
Mamífero	Mammal	Neutral	Miscellany	10
Bonito	Pretty	Positive	Positive traits	10
Comercial	Commercial	Positive	Commercial benefits	9
No común	Not common	Neutral	Ecology and biological knowledge	9
Destrucción	Destruction	Negative	Negative outcomes	9
Mata / Matar	Kill	Negative	Negative outcomes	9
Asombro	Awe	Positive	Positive feelings	9
Sorprendente	Awesome	Positive	Positive feelings	9
Dañino	Harmful	Negative	Negative traits	8
No comestible	Non-edible	Negative	Negative traits	8
Respeto	Respect	Positive	Positive feelings	8
Comestible	Edible	Positive	Commercial benefits	7
Rojo	Red	Neutral	Miscellany	7
Voraz	Voracious	Negative	Negative traits	7

S3 Table. Shark-related vocabulary (SRV). *(Continued)*

Words in Spanish	Words in English	Category	Sub-category	Frequency
Carroñero	Scavenger	Neutral	Ecology and biological knowledge	6
Océano	Ocean	Neutral	Ecology and biological knowledge	6
Escalofrío / Escalofriante	Shivers	Negative	Negative feelings	6
Mortal	Lethal	Negative	Negative feelings	6
Curiosidad	Curiosity	Positive	Positive feelings	6
Astuto	Astute	Positive	Positive traits	6
Rápido	Quick	Positive	Positive traits	6
Caro	Expensive	Positive	Commercial benefits	5
Colágeno	Collagen	Positive	Commercial benefits	5
Curativo	Healing	Positive	Commercial benefits	5
Industria	Industry	Positive	Commercial benefits	5
Pastillas	Pills	Positive	Commercial benefits	5
Altamar	High seas	Neutral	Ecology and biological knowledge	5
Nadador	Swimmer	Neutral	Ecology and biological knowledge	5
Arena	Sand	Neutral	Miscellany	5
Colmillo	Fang	Neutral	Miscellany	5
Profundidad	Deep	Neutral	Miscellany	5
Tamaño	Size	Neutral	Miscellany	5
Velocidad	Speed	Neutral	Miscellany	5
Desesperación	Despair	Negative	Negative feelings	5
Fobia	Phobia	Negative	Negative feelings	5
Emoción	Excitement	Positive	Positive feelings	5
Inteligente	Intelligent	Positive	Positive traits	5
Negocio	Business	Positive	Commercial benefits	4
Acuatico	Aquatic	Neutral	Ecology and biological knowledge	4
Inmenso	Immense	Neutral	Ecology and biological knowledge	4
Raro	Rare	Neutral	Ecology and biological knowledge	4
Atlantico	Atlantic	Neutral	Miscellany	4
Cachalote	Sperm whale	Neutral	Miscellany	4
Cetáceo	Cetacean	Neutral	Miscellany	4
China	China	Neutral	Miscellany	4
Come	Eats	Neutral	Miscellany	4
Desconocido	Unknown	Neutral	Miscellany	4
Gordo	Fat	Neutral	Miscellany	4
Angustia	Anguish	Negative	Negative feelings	4
Escapar / Huir	Escape	Negative	Negative feelings	4
Espanto	Fright	Negative	Negative feelings	4
Nervios	Nervousness	Negative	Negative feelings	4
Pavor	Dread	Negative	Negative feelings	4
Prevención	Prevention	Negative	Negative feelings	4

S3 Table. Shark-related vocabulary (SRV). *(Continued)*

Words in Spanish	Words in English	Category	Sub-category	Frequency
Suspenso	Suspense	Negative	Negative feelings	4
Heridas	Wounds	Negative	Negative outcomes	4
Cruel	Cruel	Negative	Negative traits	4
Monstruo	Monster	Negative	Negative traits	4
Asombroso	Amazing	Positive	Positive feelings	4
Hermoso / Belleza	Beautiful	Positive	Positive traits	4
Afrodisiaco	Aphrodisiac	Positive	Commercial benefits	3
Barco	Boat	Positive	Commercial benefits	3
Delicioso	Delicious	Positive	Commercial benefits	3
Exportación / Exportar	Export	Positive	Commercial benefits	3
Frito	Fried	Positive	Commercial benefits	3
Harina	Meal	Positive	Commercial benefits	3
Pescador	Fishermen	Positive	Commercial benefits	3
Turismo	Tourism	Positive	Commercial benefits	3
Vitamina	Vitamin	Positive	Commercial benefits	3
Afilado / Filoso / Filudo	Sharp	Neutral	Ecology and biological knowledge	3
Agua fria	Cold water	Neutral	Ecology and biological knowledge	3
Boca grande	Big mouth	Neutral	Ecology and biological knowledge	3
Comunicar	Communicate	Neutral	Miscellany	3
Delfin	Dolphin	Neutral	Miscellany	3
Gris	Grey	Neutral	Miscellany	3
Lejanía	Remoteness	Neutral	Miscellany	3
Orilla	Shore	Neutral	Miscellany	3
Poco	Low	Neutral	Miscellany	3
Sal	Salt	Neutral	Miscellany	3
Correr	Run!	Negative	Negative feelings	3
Llanto	Crying	Negative	Negative feelings	3
Riesgo	Risk	Negative	Negative feelings	3
Accidente	Accident	Negative	Negative outcomes	3
Dolor	Pain	Negative	Negative outcomes	3
Grito	Scream	Negative	Negative outcomes	3
Desagradable / No agradable	Unpleasant	Negative	Negative traits	3
Horrible	Horrible	Negative	Negative traits	3
Ofensivo	Offensive	Negative	Negative traits	3
Rudo	Burly	Negative	Negative traits	3
Audaz	Bold	Positive	Positive traits	3
Interesante	Interesting	Positive	Positive traits	3
Arpón	Harpoon	Positive	Commercial benefits	2
Artesanía	Hand craft	Positive	Commercial benefits	2
Carnoso	Fleshy	Positive	Commercial benefits	2
Consumo	Consumption	Positive	Commercial benefits	2
Extranjero	Foreign market	Positive	Commercial benefits	2
Fierro	Iron	Positive	Commercial benefits	2

S3 Table. Shark-related vocabulary (SRV). *(Continued)*

Words in Spanish	Words in English	Category	Sub-category	Frequency
Jugoso	Juicy	Positive	Commercial benefits	2
Lanchas	Fishing vessel	Positive	Commercial benefits	2
Protéico / Proteína	Protein	Positive	Commercial benefits	2
Red	Fishing net	Positive	Commercial benefits	2
Abundante	Abundant	Neutral	Ecology and biological knowledge	2
Grasa	Grease	Neutral	Ecology and biological knowledge	2
Grupos	Groups	Neutral	Ecology and biological knowledge	2
Hambriento	Hungry	Neutral	Ecology and biological knowledge	2
Largo	Long	Neutral	Ecology and biological knowledge	2
Mandíbula	Jaw	Neutral	Ecology and biological knowledge	2
Pequeño	Small	Neutral	Ecology and biological knowledge	2
Pesado	Heavy	Neutral	Ecology and biological knowledge	2
Suave	Soft	Neutral	Ecology and biological knowledge	2
Algas	Algae	Neutral	Miscellany	2
Anemia	Anemia	Neutral	Miscellany	2
Cine	Film	Neutral	Miscellany	2
Diamante	Diamond	Neutral	Miscellany	2
Exterminación	Extermination	Neutral	Miscellany	2
Inexistente	Non-existent	Neutral	Miscellany	2
Martillo	Hammer	Neutral	Miscellany	2
Olas	Waves	Neutral	Miscellany	2
Verano	Summer	Neutral	Miscellany	2
Vivo	Alive	Neutral	Miscellany	2
Alejarse	Distance yourself	Negative	Negative feelings	2
Auxilio	Help!	Negative	Negative feelings	2
Colera / Enojo	Anger	Negative	Negative feelings	2
Espantoso	Frightening	Negative	Negative feelings	2
Mutilación	Mutilation	Negative	Negative outcomes	2
Piernas	Legs	Negative	Negative outcomes	2
Cochino / Suciedad	Dirty	Negative	Negative traits	2
Destructor	Destroyer	Negative	Negative traits	2
Insalubre	Insalubrious	Negative	Negative traits	2
Malvado	Evil	Negative	Negative traits	2
Tirano	Tyrant	Negative	Negative traits	2
Conservación	Conserve	Positive	Positive feelings	2
Grandeza	Greatness	Positive	Positive feelings	2
Majestuoso	Majestic	Positive	Positive traits	2
Tierno / Tiernos	Cute	Positive	Positive traits	2
Unico	Unique	Positive	Positive traits	2

S3 Table. Shark-related vocabulary (SRV). *(Continued)*

Words in Spanish	Words in English	Category	Sub-category	Frequency
Barato	Cheap	Positive	Commercial benefits	1
Calcio	Calcium	Positive	Commercial benefits	1
Comercio	Commerce	Positive	Commercial benefits	1
Dinero	Money	Positive	Commercial benefits	1
Económico	Economic	Positive	Commercial benefits	1
Enlatado	Canned	Positive	Commercial benefits	1
Inversión	Investment	Positive	Commercial benefits	1
Omega 3	Omega 3	Positive	Commercial benefits	1
Pesqueria	Fishery	Positive	Commercial benefits	1
Polvo	Powder	Positive	Commercial benefits	1
Precio	Price	Positive	Commercial benefits	1
Procesado	Processed	Positive	Commercial benefits	1
Rendidor	Productive	Positive	Commercial benefits	1
Restaurante	Restaurant	Positive	Commercial benefits	1
Trabajo	Work	Positive	Commercial benefits	1
Abundancia	Abundance	Neutral	Ecology and biological knowledge	1
Aspero	Rough	Neutral	Ecology and biological knowledge	1
Cadena alimenticia	Food web	Neutral	Ecology and biological knowledge	1
Cardumen	Schooling	Neutral	Ecology and biological knowledge	1
Ciego	Blind	Neutral	Ecology and biological knowledge	1
Contaminación	Pollution	Neutral	Ecology and biological knowledge	1
Escamas	Scales	Neutral	Ecology and biological knowledge	1
Escualo	Shark	Neutral	Ecology and biological knowledge	1
Fondo	Benthic	Neutral	Ecology and biological knowledge	1
Liso	Smooth	Neutral	Ecology and biological knowledge	1
Necesario	Necessary	Neutral	Ecology and biological knowledge	1
Ojasos	Big eyes	Neutral	Ecology and biological knowledge	1
Ojos	Eyes	Neutral	Ecology and biological knowledge	1
Piel dura	Hard skin	Neutral	Ecology and biological knowledge	1
Reciclador	Recycler	Neutral	Ecology and biological knowledge	1
Solitario	Solitary	Neutral	Ecology and biological knowledge	1

S3 Table. Shark-related vocabulary (SRV). *(Continued)*

Words in Spanish	Words in English	Category	Sub-category	Frequency
Solo	Lonely	Neutral	Ecology and biological knowledge	1
Toxinas	Toxins	Neutral	Ecology and biological knowledge	1
Variedad	Diversity	Neutral	Ecology and biological knowledge	1
Vida larga	Long-lived	Neutral	Ecology and biological knowledge	1
Acción	Action	Neutral	Miscellany	1
Agua dulce	Freshwater	Neutral	Miscellany	1
Agudo	Acute	Neutral	Miscellany	1
Artritis	Arthritis	Neutral	Miscellany	1
Asia	Asia	Neutral	Miscellany	1
Aumenta	Increase	Neutral	Miscellany	1
Australia	Australia	Neutral	Miscellany	1
Bacalao	Cod	Neutral	Miscellany	1
Calentamiento global	Global warming	Neutral	Miscellany	1
Cálido	Warm	Neutral	Miscellany	1
Calor	Heat	Neutral	Miscellany	1
Cantidad	Quantity	Neutral	Miscellany	1
Caribe	Caribbean	Neutral	Miscellany	1
Celeste	Light blue	Neutral	Miscellany	1
Clima	Weather	Neutral	Miscellany	1
Contrabando	Smuggling	Neutral	Miscellany	1
Coral	Coral	Neutral	Miscellany	1
Cornudo	Horns	Neutral	Miscellany	1
Cuchillo	Knife	Neutral	Miscellany	1
Desmenuzado	Crumbled	Neutral	Miscellany	1
Dorado	Golden	Neutral	Miscellany	1
Duro	Hard	Neutral	Miscellany	1
EEUU	USA	Neutral	Miscellany	1
Espada	Sword	Neutral	Miscellany	1
Espinas	Spines	Neutral	Miscellany	1
Esquelético	Skeletal	Neutral	Miscellany	1
Extension	Broad	Neutral	Miscellany	1
Fósiles	Fossils	Neutral	Miscellany	1
Gato	Cat	Neutral	Miscellany	1
Gente	People	Neutral	Miscellany	1
Habilidad	Skills	Neutral	Miscellany	1
Historietas	Stories	Neutral	Miscellany	1
Hombre	Man	Neutral	Miscellany	1
Huesudo	Bony	Neutral	Miscellany	1
Invasion	Invasion	Neutral	Miscellany	1
Japón	Japan	Neutral	Miscellany	1
Limitado consumo	Limited consumption	Neutral	Miscellany	1
Lobos marinos	Sea lions	Neutral	Miscellany	1
Mal uso	Misuse	Neutral	Miscellany	1

S3 Table. Shark-related vocabulary (SRV). *(Continued)*

Words in Spanish	Words in English	Category	Sub-category	Frequency
Natural	Natural	Neutral	Miscellany	1
Naturaleza	Nature	Neutral	Miscellany	1
Noche	Night	Neutral	Miscellany	1
Normal	Normal	Neutral	Miscellany	1
Nuevo	New	Neutral	Miscellany	1
Nunca visto	Never seen	Neutral	Miscellany	1
Obesidad	Obesity	Neutral	Miscellany	1
Rebelde	Rebel	Neutral	Miscellany	1
Sin espinas	Boneless	Neutral	Miscellany	1
Submarino	Submarine	Neutral	Miscellany	1
Tablista	Surfer	Neutral	Miscellany	1
Tragar	Swallow	Neutral	Miscellany	1
Vida	Life	Neutral	Miscellany	1
Videos	Videos	Neutral	Miscellany	1
Volumen	Volume	Neutral	Miscellany	1
Alarmante	Alarming	Negative	Negative feelings	1
Ansiedad	Anxiety	Negative	Negative feelings	1
Arriesgado	Risky	Negative	Negative feelings	1
Asco	Disgust	Negative	Negative feelings	1
Asqueroso	Disgusting	Negative	Negative feelings	1
Asustado	Scared	Negative	Negative feelings	1
Asustar	Frighten	Negative	Negative feelings	1
Atento	Alertness	Negative	Negative feelings	1
Aterrador	Scary	Negative	Negative feelings	1
Decepción	Disappointment	Negative	Negative feelings	1
Defensa	Defense	Negative	Negative feelings	1
Desgracia	Misfortune	Negative	Negative feelings	1
Furia	Fury	Negative	Negative feelings	1
Injusto	Unfair	Negative	Negative feelings	1
Inseguridad	Insecurity	Negative	Negative feelings	1
Lágrimas	Tears	Negative	Negative feelings	1
Pena	Sadness	Negative	Negative feelings	1
Pesadillas	Nightmares	Negative	Negative feelings	1
Piedad	Mercy please!	Negative	Negative feelings	1
Trágico	Tragic	Negative	Negative feelings	1
Vértigo	Vertigo	Negative	Negative feelings	1
Amputacion	Amputation	Negative	Negative outcomes	1
Desgarro	Tear	Negative	Negative outcomes	1
Enfermedades	Diseases	Negative	Negative outcomes	1
Movilizarse	Displaced	Negative	Negative outcomes	1
Rompe mallas	Mesh-breaker	Negative	Negative outcomes	1
Triturar	Crush	Negative	Negative outcomes	1
Voltea botes	Turn boats	Negative	Negative outcomes	1
Amargo	Bitter	Negative	Negative traits	1
Canival	Cannibal	Negative	Negative traits	1
Desabrido	Tasteless	Negative	Negative traits	1
Desalmado	Fiend	Negative	Negative traits	1

S3 Table. Shark-related vocabulary (SRV). *(Continued)*

Words in Spanish	Words in English	Category	Sub-category	Frequency
Desastrozo	Disastrous	Negative	Negative traits	1
Desmesurado	Excessive	Negative	Negative traits	1
Despiadado	Ruthless	Negative	Negative traits	1
Dificil de pescar	Hard to fish	Negative	Negative traits	1
Enemigo	Enemy	Negative	Negative traits	1
Extrangulador	Strangler	Negative	Negative traits	1
Impuro	Impure	Negative	Negative traits	1
Macabro	Macabre	Negative	Negative traits	1
Molesto	Angry	Negative	Negative traits	1
No saludable	Unhealthy	Negative	Negative traits	1
Perverso	Perverse	Negative	Negative traits	1
Poco amigable	Not friendly	Negative	Negative traits	1
Problemático	Problematic	Negative	Negative traits	1
Rabioso	Mad	Negative	Negative traits	1
Sigiloso	Sneaky	Negative	Negative traits	1
Violento	Violent	Negative	Negative traits	1
Admiración	Admiration	Positive	Positive feelings	1
Atracción	Attraction	Positive	Positive feelings	1
Autoridad	Authority	Positive	Positive feelings	1
Divertido	Fun	Positive	Positive feelings	1
Euforia	Euphoria	Positive	Positive feelings	1
Impresivo	Impressive	Positive	Positive feelings	1
Libertad	Freedom	Positive	Positive feelings	1
Paz	Peace	Positive	Positive feelings	1
Poder	Power	Positive	Positive feelings	1
Agil	Agile	Positive	Positive traits	1
Bueno	Good	Positive	Positive traits	1
Chevere	Cool	Positive	Positive traits	1
Dulce	Sweet	Positive	Positive traits	1
Especial	Special	Positive	Positive traits	1
Espectacular	Spectacular	Positive	Positive traits	1
Extraordinario	Extraordinary	Positive	Positive traits	1
Fenomenal	Phenomenal	Positive	Positive traits	1
Guardián	Guardian	Positive	Positive traits	1
Impresionante	Spectacular	Positive	Positive traits	1
Limpieza	Clean	Positive	Positive traits	1
Lindo	Nice	Positive	Positive traits	1
Listo	Smart	Positive	Positive traits	1
