# Supplementary material for: Attitudes and misconceptions towards sharks and shark meat consumption along the Peruvian coast
Source: PLoS One. 2018 Aug 29;13(8):e0202971. doi: 10.1371/journal.pone.0202971 (PMC6114843; doi:10.1371/journal.pone.0202971)
Supplement: S4 Table — (PDF) [file pone.0202971.s004.pdf]

S4 Table. Total fish landings and shark landings caught by the small-scale fishing fleets of Peru. Landings data collected by the Peruvian Institute of the Sea (IMARPE) on multiple monitored sites was aggregated by coastal Region.

Region	Monitored landing sites	Taxa	2013	2014	2015	Total
Tumbes	Puerto Pizarro and Zorritos	All fishes (tonnes)	4161	5431	4492	14084
		Sharks (tonnes)	34	26	14	74
		Sharks (%)	0.81%	0.48%	0.31%	**0.52%**
Piura	Las Delicias, Paita, Parachique, Puerto Rico and Talara	All fishes (tonnes)	58371	42053	34929	135353
		Sharks (tonnes)	165	101	148	413
		Sharks (%)	0.28%	0.24%	0.42%	**0.31%**
Lambayeque	San José	All fishes (tonnes)	2136	4535	11838	18509
		Sharks (tonnes)	90	130	186	405
		Sharks (%)	4.20%	2.86%	1.57%	**2.19%**
La Libertad	Salaverry	All fishes (tonnes)	1992	2933	2751	7677
		Sharks (tonnes)	565	552	627	1745
		Sharks (%)	28.37%	18.82%	22.81%	**22.73%**
Ancash	Chimbote	All fishes (tonnes)	11109	9541	6974	28434
		Sharks (tonnes)	42	55	56	153
		Sharks (%)	0.38%	0.57%	0.81%	**0.54%**
Lima	Callao, Huacho and Pucusana	All fishes (tonnes)	18176	17171	16973	52320
		Sharks (tonnes)	372	247	338	957
		Sharks (%)	2.05%	1.44%	1.99%	**1.83%**
Ica	Laguna Grande and San Andrés	All fishes (tonnes)	12292	14736	11630	38658
		Sharks (tonnes)	0	2	1	3
		Sharks (%)	0.00%	0.01%	0.01%	**0.01%**
Arequipa	Atico, La Planchada and Matarani	All fishes (tonnes)	6407	8076	5894	20377
		Sharks (tonnes)	62	61	90	212
		Sharks (%)	0.96%	0.75%	1.53%	**1.04%**
Moquegua	Ilo	All fishes (tonnes)	6563	8898	6084	21545
		Sharks (tonnes)	1291	2006	2436	5733
		Sharks (%)	19.67%	22.54%	40.04%	**26.61%**
Tacna	Morro Sama	All fishes (tonnes)	1441	4398	1880	7719
		Sharks (tonnes)	31.897	30.865	70.496	133.258
		Sharks (%)	2.21%	0.70%	3.75%	**1.73%**
