# Supplementary material for: Attitudes and misconceptions towards sharks and shark meat consumption along the Peruvian coast
Source: PLoS One. 2018 Aug 29;13(8):e0202971. doi: 10.1371/journal.pone.0202971 (PMC6114843; doi:10.1371/journal.pone.0202971)

S1 Fig. Proportion of shark meat consumers per city. (A) Consumers who eat sharks under the 'Tiburon' (grey bars) and 'Tollo' (blue bars) common names, and (B) 'Tollo' consumers who are aware that 'Tollo' is a generic name for sharks (i.e. 'Conscious' shark meat consumers).

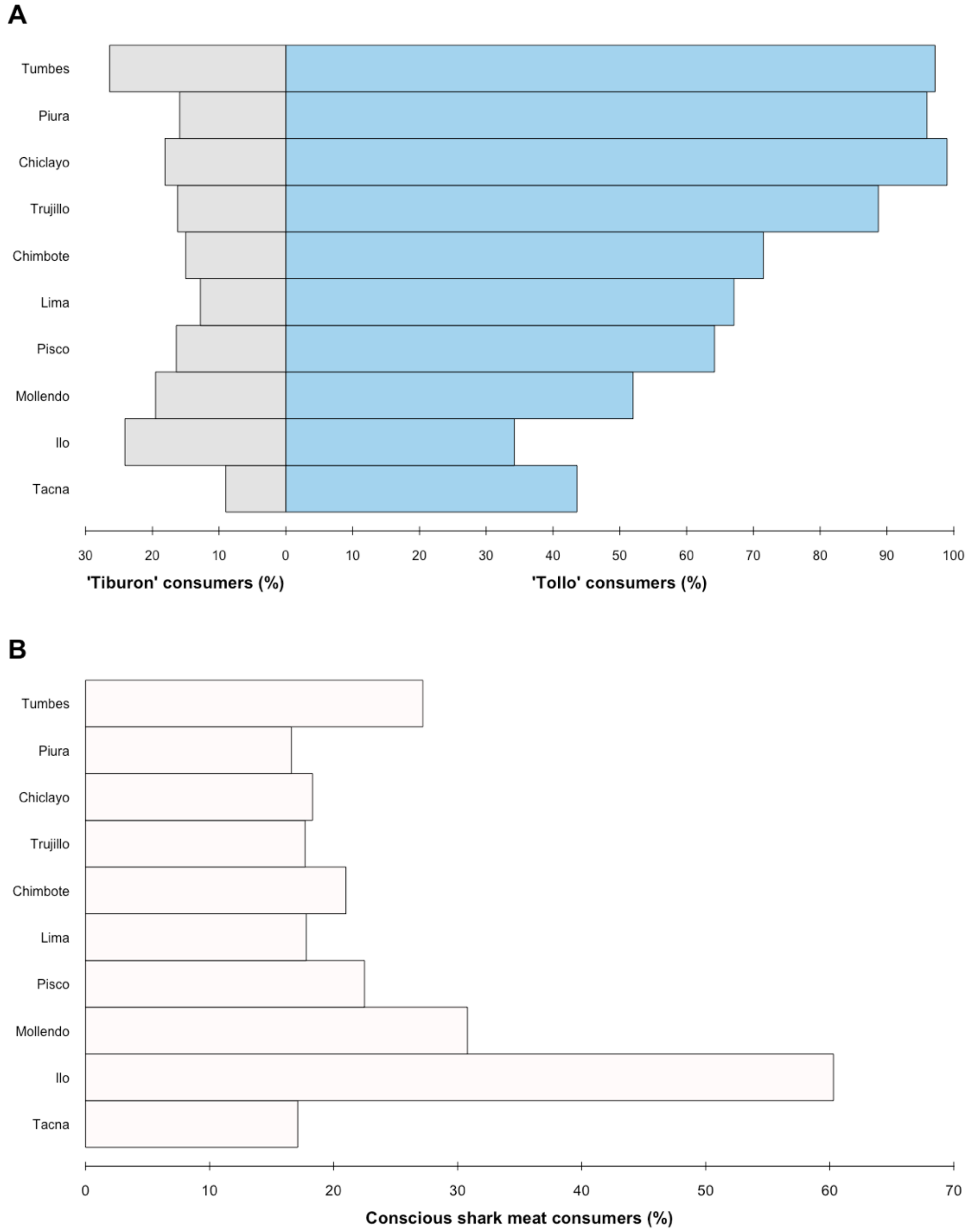

Supplement: S1 Fig — (A) Consumers who eat sharks under the ‘Tiburon’ (grey bars) and ‘Tollo’ (blue bars) common names, and (B) ‘Tollo’ consumers who are aware that ‘Tollo’ is a generic name for sharks (i.e., ‘Conscious’ shark meat consumers). (PDF) [file pone.0202971.s005.pdf]
