# Supplementary material for: Attitudes and misconceptions towards sharks and shark meat consumption along the Peruvian coast
Source: PLoS One. 2018 Aug 29;13(8):e0202971. doi: 10.1371/journal.pone.0202971 (PMC6114843; doi:10.1371/journal.pone.0202971)

S2 Fig. Distribution of 'tollo' consumers per city as a function of their frequency of shark meat consumption. Shark meat consumers were categorized into: Regular consumers (REG), Occasional consumers (OCC), Unusual consumers (UNU) and Former consumers (FOR)

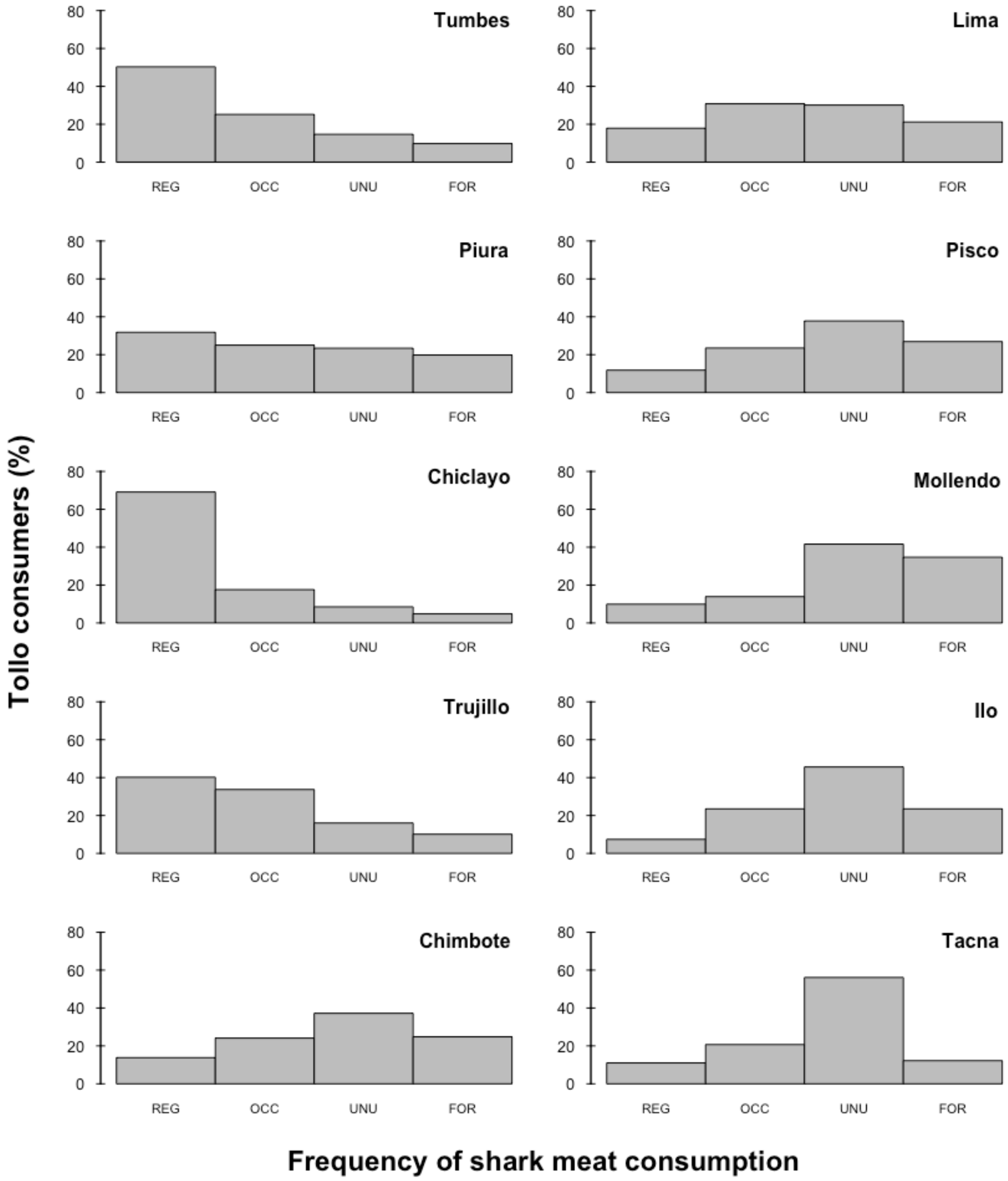

Supplement: S2 Fig — Shark meat consumers were categorized into: Regular consumers (REG), Occasional consumers (OCC), Unusual consumers (UNU) and Former consumers (FOR) (PDF) [file pone.0202971.s006.pdf]
