# Supplementary material for: Attitudes and misconceptions towards sharks and shark meat consumption along the Peruvian coast
Source: PLoS One. 2018 Aug 29;13(8):e0202971. doi: 10.1371/journal.pone.0202971 (PMC6114843; doi:10.1371/journal.pone.0202971)

S3 Fig. Proportion of the surveyed population per city that knows that sharks are present, maybe present, or are not present in Peruvian waters. The proportion of participants that did not answer this question are included in the N/A category.

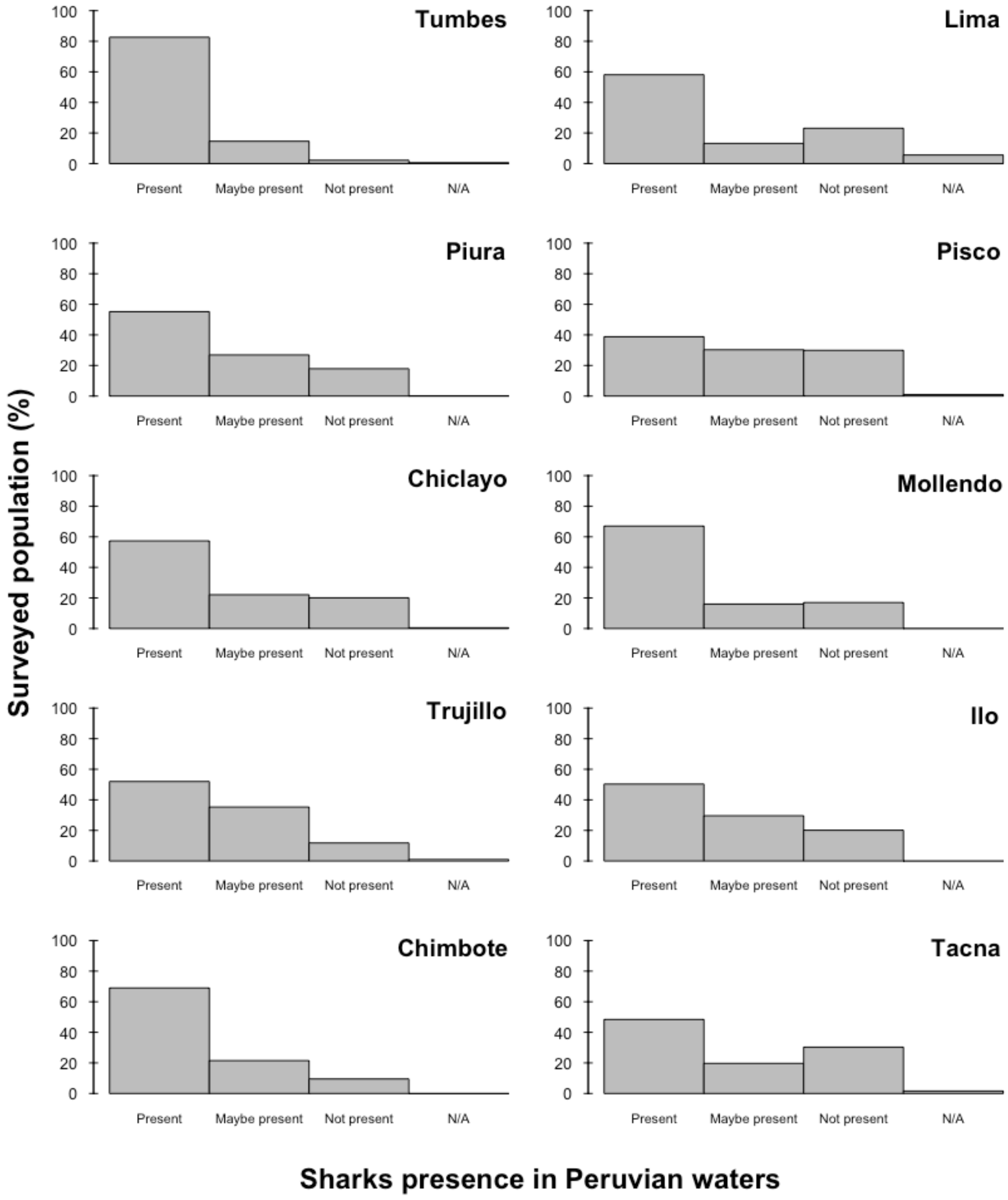

Supplement: S3 Fig — The proportion of participants that did not answer this question are included in the N/A category. (PDF) [file pone.0202971.s007.pdf]
