# Supplementary material for: Attitudes and misconceptions towards sharks and shark meat consumption along the Peruvian coast
Source: PLoS One. 2018 Aug 29;13(8):e0202971. doi: 10.1371/journal.pone.0202971 (PMC6114843; doi:10.1371/journal.pone.0202971)

S4 Fig. Capacity to mention shark names by participants who ascertained that sharks are present in Peruvian waters, segregated city. N references the total number of mentions, μ references the average number of words mentioned per city and σ references its standard deviation.

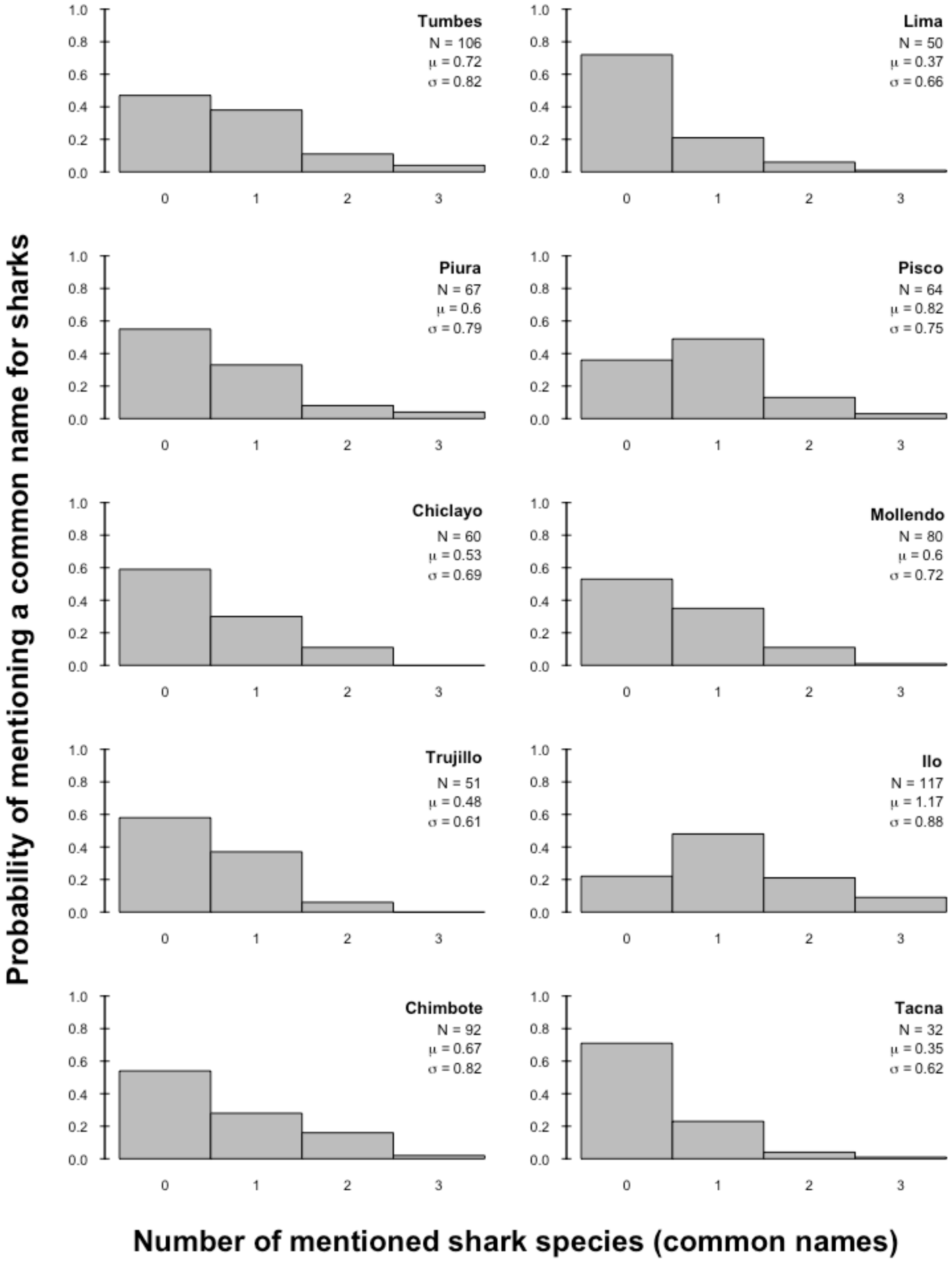

Supplement: S4 Fig — N references the total number of mentions, μ references the average number of words mentioned per city and σ references its standard deviation. (PDF) [file pone.0202971.s008.pdf]
