# Supplementary material for: Attitudes and misconceptions towards sharks and shark meat consumption along the Peruvian coast
Source: PLoS One. 2018 Aug 29;13(8):e0202971. doi: 10.1371/journal.pone.0202971 (PMC6114843; doi:10.1371/journal.pone.0202971)

S6 Fig. Distribution of the Individual Attitude Scores per city. μ references the average attitude score per city and σ references its standard deviation.

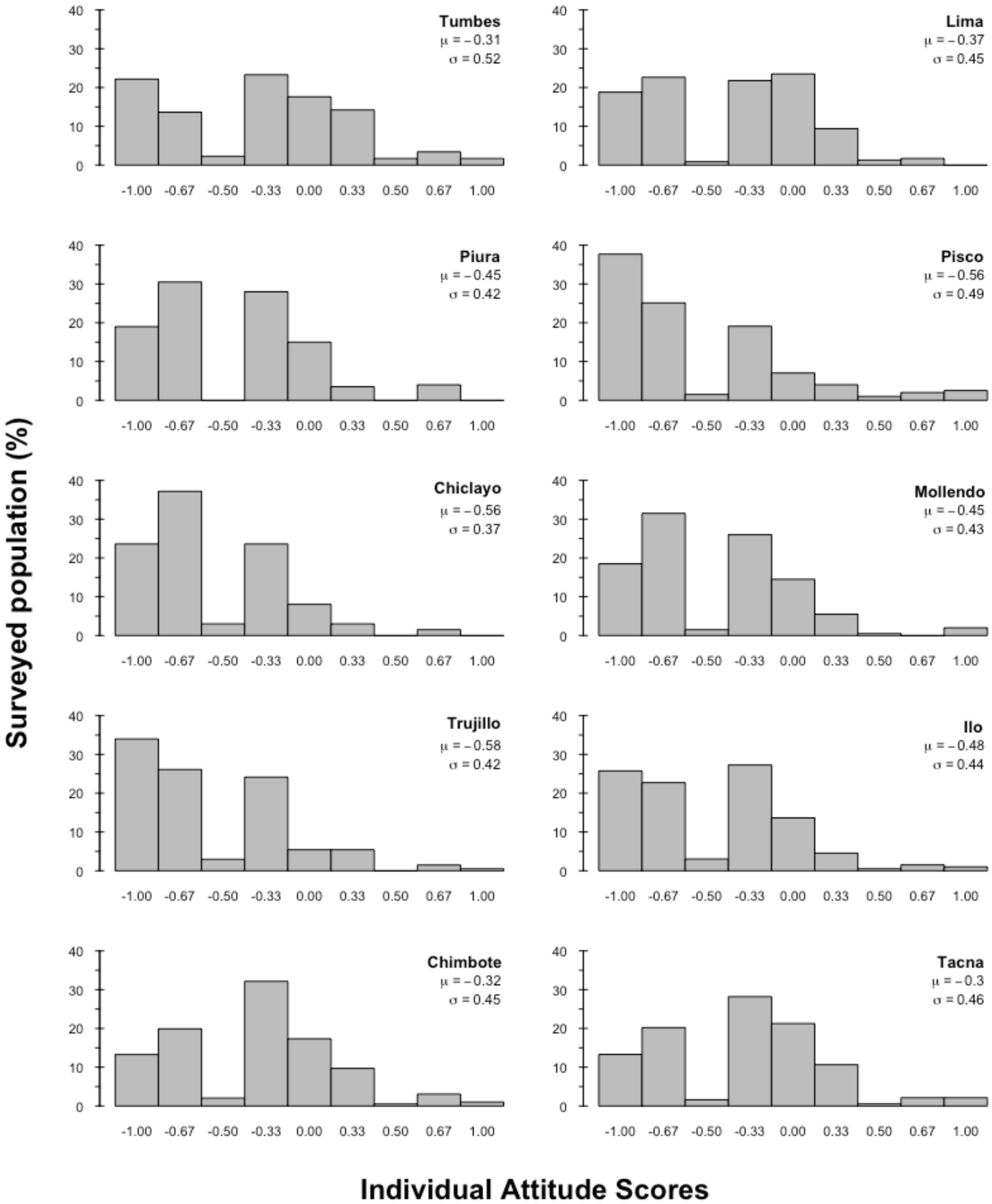

Supplement: S6 Fig — μ references the average attitude score per city and σ references its standard deviation. (PDF) [file pone.0202971.s010.pdf]
