# Supplementary material for: Attitudes and misconceptions towards sharks and shark meat consumption along the Peruvian coast
Source: PLoS One. 2018 Aug 29;13(8):e0202971. doi: 10.1371/journal.pone.0202971 (PMC6114843; doi:10.1371/journal.pone.0202971)

S5 Fig. Radar plots showing the relative importance of the eight different categories that constitute each city's Attitude Profile. Each radar consists of four concentric octagons that extend from the origin. Each level denotes a 10% increase in the frequency of the words per category, where the origin marks a score of 0% and the outermost octagon of 40%. Acronyms in the figure stand for: BH: Benefits to humans; M: Miscellaneous; NT: Negative traits; NO: Negative outcomes of human-shark interactions; NF: Negative feelings; EBK: Ecological and biological knowledge; PT: Positive traits; PF: Positive feelings.

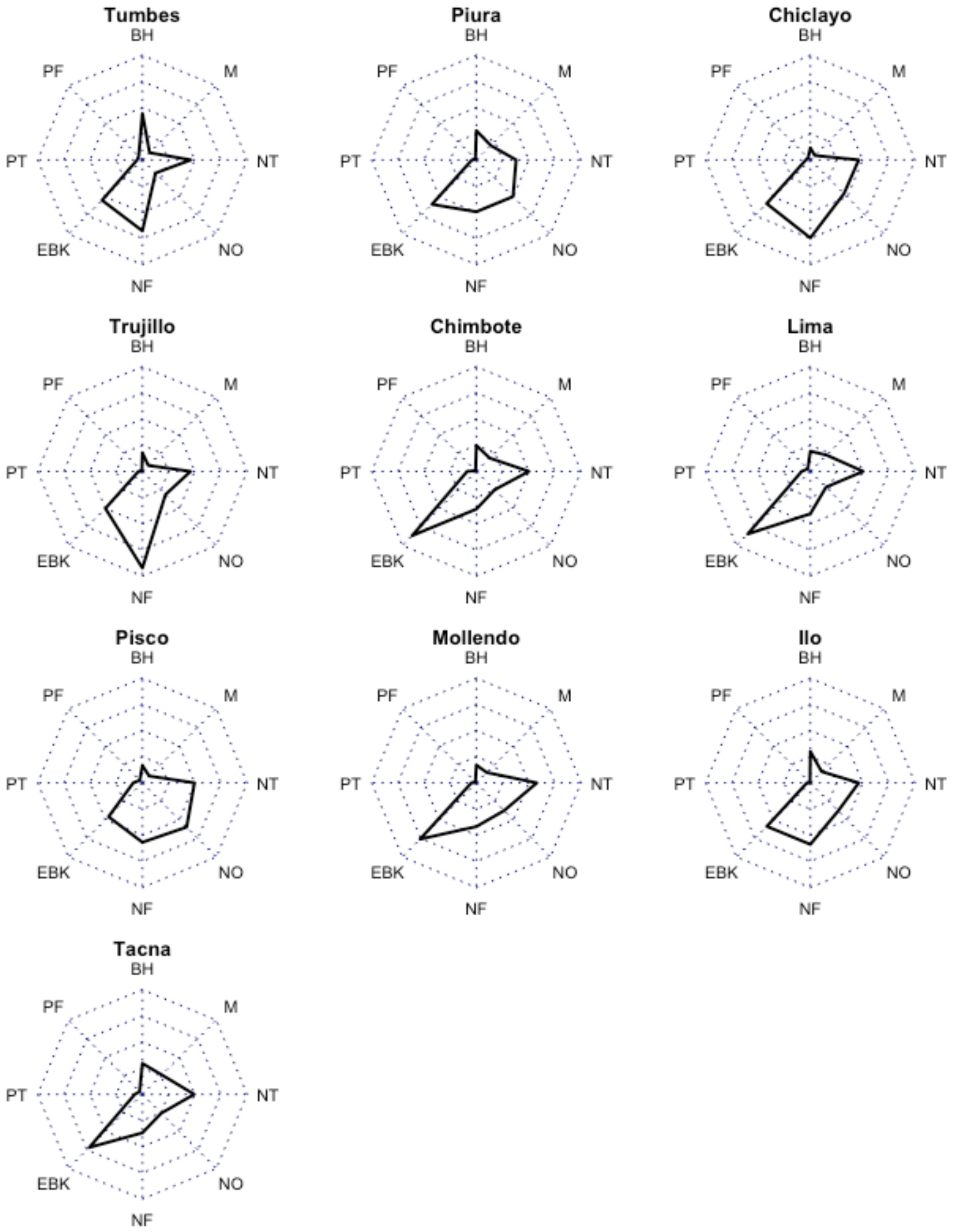

Supplement: S5 Fig — Each radar consists of four concentric octagons that extend from the origin. Each level denotes a 10% increase in the frequency of the words per category, where the origin marks a score of 0% and the outermost octagon of 40%. Acronyms in the figure stand for: BH: Benefits to humans; M: Miscellaneous; NT: Negative traits; NO: Negative outcomes of human-shark interactions; NF: Negative feelings; EBK: Ecological and biological knowledge; PT: Positive traits; PF: Positive feelings. (PDF) [file pone.0202971.s009.pdf]
